# The different pathogeneses of sporadic adenoma and adenocarcinoma in non-ampullary lesions of the proximal and distal duodenum

**DOI:** 10.18632/oncotarget.17051

**Published:** 2017-04-12

**Authors:** Ayumi Niwa, Seiya Kuwano, Hiroyuki Tomita, Keita Kimura, Yukiya Orihara, Tomohiro Kanayama, Kei Noguchi, Kenji Hisamatsu, Takayuki Nakashima, Yuichiro Hatano, Akihiro Hirata, Tatsuhiko Miyazaki, Kazuhiro Kaneko, Takuji Tanaka, Akira Hara

**Affiliations:** ^1^ Department of Tumor Pathology, Gifu University Graduate School of Medicine, Gifu, Japan; ^2^ Division of Animal Experiment, Life Science Research Center, Gifu University, Gifu, Japan; ^3^ Division of Pathology, Gifu University Hospital, Gifu, Japan; ^4^ Department of Gastroenterology, Endoscopy Division, National Cancer Center Hospital East, Kashiwa, Japan; ^5^ Department of Diagnostic Pathology (DDP) and Research Center of Diagnostic Pathology (RC-DiP), Gifu Municipal Hospital, Gifu, Japan

**Keywords:** duodenal neoplasms, beta-catenin

## Abstract

Non-ampullary duodenal adenoma with activation of Wnt/β-catenin signalling is common in familial adenomatous polyposis (FAP) patients, whereas sporadic non-ampullary adenoma is uncommon. The adenoma-carcinoma sequence similar to colon cancer is associated with duodenal tumors in FAP, but not always in sporadic tumors. We obtained 37 non-ampullary duodenal tumors, including 25 adenomas and 12 adenocarcinomas, were obtained from biopsies and endoscopic resections. We performed immunohistochemistry for β-catenin, the hallmark of Wnt activation, and aldehyde dehydrogenase 1 (ALDH1), a putative cancer stem cell marker. In non-ampullary lesions, abnormal nuclear localization of β-catenin was observed in 21 (84.0%) of 25 adenomas and 4 (33.3%) of 12 adenocarcinomas. In the proximal duodenum, nuclear β-catenin was less frequent in both adenomas and adenocarcinomas. Gastric duodenal metaplasia (GDM) was observed only in the proximal duodenum. All adenomas with GDM were the gastric foveolar and pyloric gland types, and showed only membranous β-catenin. The intestinal-type adenomas had nuclear β-catenin in the proximal and distal duodenum. ALDH1-positive cells were more frequent in adenocarcinomas than adenomas. Nuclear β-catenin accumulation frequently occurred in ALDH1-positive cells in adenoma, but not in adenocarcinoma. In the non-ampullary proximal duodenum, Wnt/β-catenin pathway activation was more closely associated with adenomas than adenocarcinomas, and while it might cooperate with ALDH1 in adenoma, it does not in adenocarcinoma. The pathogenesis thus may differ between sporadic adenoma and adenocarcinoma of non-ampullary duodenal lesions, especially in the proximal and distal duodenum.

## INTRODUCTION

Most small intestine adenomas, including the ampulla of Vater, develop in the duodenum. Among these, sporadic non-ampullary (except the ampulla of Vater) duodenal adenomas are uncommon [1, 2] compared to adenomas in familial adenomatous polyposis (FAP), with a germline mutation in the adenomatous polyposis coli (*APC*) gene. Approximately 60% of duodenal adenomas develop in patients with FAP, while the remaining 40% are sporadic [3]. Non-ampullary duodenal adenoma is thought to be a precancerous lesion, and the model of the adenoma-carcinoma sequence is predicted to involve the small intestine, including the duodenum and colorectum [4–6].

The Wnt/β-catenin pathway promotes organ development and tissue homeostasis [7], and disruption of the pathway causes various malignancies [8]. β-catenin, which binds to the product of the *APC* gene, is a multifunctional cytoplasmic protein with a molecular weight of 92 kDa [9]. The accumulation of β-catenin in the nucleus is the hallmark of Wnt pathway activation, and leads to cell proliferation by activating lymphoid enhancer factor/T-cell factor transcription factors [7]. Wnt pathway activation, particularly by *APC* and *CTNNB1* gene mutation, is associated with early events in colon carcinogenesis in the model of the adenoma-carcinoma sequence [9]. Nuclear β-catenin translocation increased with the progression of colorectal tissue from normal epithelial tissue, adenomas, to carcinomas in the colorectum [10].

The aldehyde dehydrogenase (ALDH) superfamily constitutes a group of NAD(P)^+^-dependent enzymes that metabolize endogenous and exogenous aliphatic and aromatic aldehyde molecules by oxidation to their corresponding carboxylic acids [13]. The human genome includes 19 known *ALDH* genes [14]. ALDH1 is a cytoplasmic member of the ALDH family of enzymes. ALDH1 expression was originally detected in a small subpopulation of hematopoietic stem/progenitor cells [15, 16]. ALDH1 is thought to be a putative cancer stem cell marker in the stomach and colorectal cancers [17, 18].

In the majority of colorectal cancers, the Wnt/β-catenin pathway is necessary for the self-renewal of cancer stem cells (CSCs) [19, 20]. On the other hand, ALDH1 is a putative CSC marker, and ALDH1-positive cells have CSC potential in colorectal and gastric cancers [17, 21–23]. ALDH1-positive cells have shown higher levels of the regulatory components of Wnt/β-catenin signalling, such as β-catenin and TCF-4, compared with ALDH1-negative cells in breast cancer [24].

Gastric duodenal metaplasia (GDM) is a histologically detectable mucosal change thought to evolve following *Helicobacter pylori* infection, celiac disease, or Crohn's disease [25–27]. The regenerative stimuli are caused by an aberrant high production of gastric acid triggered by *Helicobacter pylori* infection [28]. GDM has been found even in the absence of these conditions and in duodenal adenomas [29]. Non-ampullary adenoma/carcinoma more often develops on the oral (proximal) side of the ampulla in Japan because of the higher rate of *H. pylori* infection compared with that in Western countries [30]. Further, the anal (distal) side of non-ampullary adenoma/carcinoma frequently occurs with colon adenoma/carcinoma, which may be influenced by bile acids [30].

In this study, we evaluated β-catenin and ALDH1 expression in non-ampullary duodenal adenomas and adenocarcinomas using immunohistochemistry (IHC), and investigated the clinicopathological characteristics and non-ampullary duodenal carcinogenesis to provide a foundation for therapy development.

## RESULTS

### Case pathology

Thirty-seven patients were included in this study (Table 1), 29 of which were male and 8 were female. A total of 25 sporadic non-ampullary duodenal adenomas (25 patients) and 12 sporadic non-ampullary duodenal adenocarcinomas (9 patients) were obtained during the study period. Patient ages ranged from 26 to 77 years (mean, 40.5 years). The tumor position in the duodenum, except the ampulla of Vater, was separated into two parts. The upper part (proximal) was from the bulb to the periampulla, while the lower part (distal) was from the periampulla to the descending duodenum. Most adenocarcinomas were detected in the upper part (9 of 12 cases) because all adenocarcinoma cases were resected using the limiting technique, i.e. EMR/ESD. Biopsy was performed on 16 lesions (43.2%) and EMR/ESD was performed on 21 lesions (56.8%). Tumor size ranged from 4 to 70 mm (mean, 37.0 mm), and the tumor size of adenocarcinoma patients was higher than that of adenoma patients (*P* < 0.05). Additionally, no patients had a family history of Lynch syndrome.

**Table 1:** Patient characteristics

Characteristic	Adenoma, n(%)	Carcinoma, n(%)	Total, n(%)
Total no. of patients	25 (67.6)	12 (22.4)	37 (100)
Age, yr	62.4 ± 13.4 (26-75)	70.2 ± 7.4 (61-77)	66.3 ± 4.3 (26-77)
Sex			
Female	6 (75)	2 (25)	8 (100)
Male	20 (58.1)	9 (25.8)	29 (100)
Total no. of lesions	25 (67.6)	12 (29.4%)	37 (100)
Method			
Biopsy	16 (100)	0 (0)	16 (100)
EMR or ESD	10 (47.6)	11 (52.4)	21 (100)
Tumor position in duodenum			
Upper part (Bulb to periampulla)	16 (64)	9 (36)	25 (100)
Lower part (Periampulla to descending)	9 (75)	3 (25)	12 (100)
Tumor size (largest diameter, mm)	17.8 ± 9.8 (4-37)	29.5 ± 17.5(6-70)*	23.7 ± 5.4 (4-70)

Data are presented as average ± SD (mean range).

EMR, endoscopic mucosal resection; ESD, endoscopic submucosal dissection.

*P < 0.05: adenoma versus adenocarcnoma in the tumor size

### Nuclear β-catenin expression was frequent in adenoma but not adenocarcinoma in the non-ampullary duodenum

**Figure 1:**
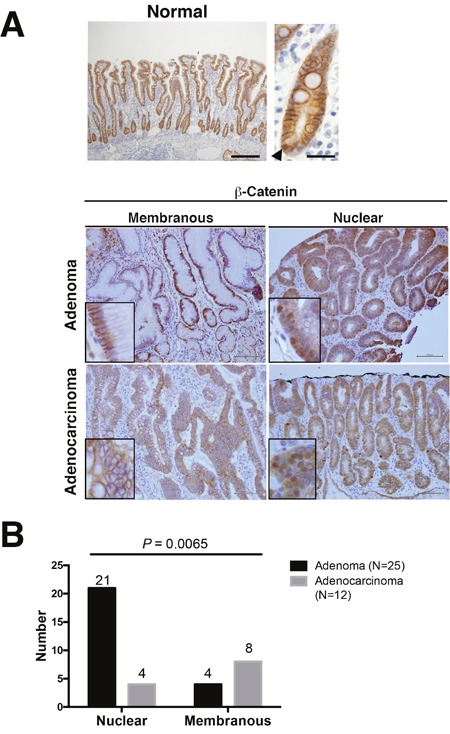
β-Catenin expression in adenoma and adenocarcinoma of the non-ampullary duodenum **(A)** Representative photos of membranous and nuclear β-catenin expression in normal mucosa, adenoma, and adenocarcinoma. The upper panel (*left*) indicates a normal epithelium with a nuclear β-catenin positive cell. Insets in adenomas and adenocarcinomas: enlarged photos at the high-power field in each photo. Bars: 100 μm. **(B)** Number of nuclear or membranous expression of β-catenin in adenomas and adenocarcinomas.

To investigate the expression of β-catenin in non-ampullary duodenal adenoma and adenocarcinoma, we performed IHC with a β-catenin antibody. Normal epithelium in the duodenum showed strong membranous staining for β-catenin with a few nuclear β-catenin-positive cell in the bottom, and aberrant expression was observed in both adenoma and adenocarcinoma (Figure 1A and Supplementary Figure 1). Abnormal nuclear localization of β-catenin was observed in 21 (86.4%) of 25 adenomas, which was higher than the 4 (22.2%) observed in 12 adenocarcinomas (Figure 1B). These data suggest that Wnt/β-catenin activation is more closely associated with adenoma compared with adenocarcinoma in non-ampullary lesions.

Non-ampullary duodenal adenomas with high-grade dysplasia and large size (≥20 mm in diameter) indicate a high risk of progression to adenocarcinoma [31]. In our cohort, 14 (56%) of 25 adenomas were more than 20 mm at their largest diameter, and nuclear β-catenin was frequent (93%) in these large adenomas (≥20 mm) (Table 2). These results indicate that Wnt/β-catenin activation may contribute to the progression of sporadic non-ampullary duodenal adenoma similar to colorectal carcinogenesis. On the other hand, the relationship between nuclear β-catenin, i.e., Wnt activation, and tumor size may be associated with aberrant apoptosis of tumor cells. This may be supported by previous reports nuclear β-catenin contributes to increased tumor size [32, 33]. Nuclear β-catenin is less frequent in adenocarcinoma than in adenoma in non-ampullary lesions. This suggests the conventional adenoma-carcinoma sequence associated with activation of Wnt/β-catenin pathway may be less frequent in non-ampullary lesions compared with colon carcinogenesis.

**Table 2:** β-Catenin expression in non-ampullary adenoma (n=25)

	β-Catenin expression			
	Nuclear/cytoplasmic	Membranous		
Tumor size (largest diameter, mm)				
≥ 20mm	13 (13%)	1 (7%)	14 (56%)	
< 20mm	3 (27%)	8 (73%)	11 (44%)	*P*=0.0021 ^a^
GDM (+)	0	4 (16%)	4 (16%)	
GDM (-)	21(84%)	0	21 (84%)	*P*< 0.001 ^a^

GDM, Gastroduodenal metaplasia

a) Nuclear/cytoplasmic versus membranous staining in β-catenin

### GDM was observed only in gastric type adenoma with membranous β-catenin

**Figure 2:**
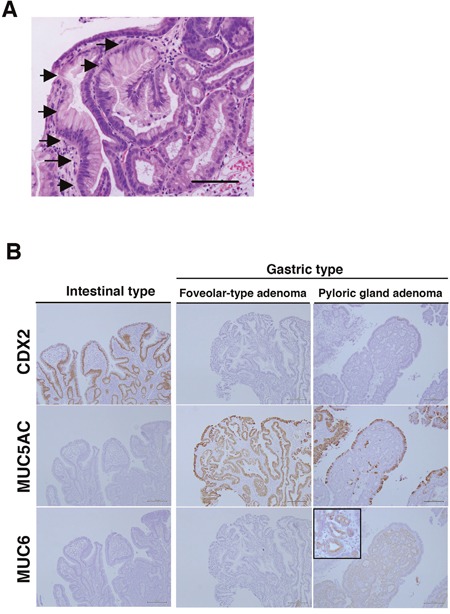
Gastric duodenal metaplasia (GDM) and histological types of duodenal adenomas in non-ampullary lesions **(A)** Representative GDM in the superficial epithelium of non-ampullary adenoma. Arrows indicate the gastric foveolar epithelium-like lesions with GDM. Bars: 200 μm. **(B)** CDX2, MUC5AC, and MUC6 expressions in intestinal-type and gastric type, i.e., foveolar-type, pyloric gland, adenoma. Inset: enlarged photo at the high-power field. Bars: 200 μm.

We focused on the positional differences and histological subtypes of adenomas and adenocarcinomas of non-ampullary duodenal lesions. In the initial manifestation of GDM of our cohort, small patches of gastric foveolar cells appear near the tip of a villus (Figure 2A). The patchy GDM in the duodenal mucosa indicates that GDM might be a reactive metaplasia rather than a congenital lesion. GDM was found in 4 (16%) of 25 adenomas and all the cases were located in the proximal duodenum. It follows that GDM is commonly observed in the proximal duodenum and is associated with peptic ulceration caused by *Helicobacter pylori* infection in most cases [34].

To clarify what types of adenomas are associated with GDM, we performed IHC for CDX-2 (a marker of intestinal origin), MUC5AC (a marker of gastric foveolar mucin), and MUC6 (a marker of gastric pyloric gland mucin). All 4 cases with GDM were gastric type adenomas. Two foveolar-type adenomas showed CDX-2(-), MUC5AC(+), MUC6(-), and 2 pyloric gland adenomas showed CDX-2(-), MUC5AC(-), MUC6(+) (Figure 2B). GDM is frequently observed in gastric type adenoma and/or heterotopic gastric mucosa in the duodenum [35], and β-catenin expression is almost only in the membrane, but not the nucleus [36].

All 4 of our cases showed only membranous β-catenin expression. The other 21 adenomasshowed nuclear β-catenin expression and these were all intestinal-type adenomas identified with expressions of CDX2(+), MUC5AC(-), and MUC6(-) (Table 2). These data suggest that activation of the Wnt/β-catenin pathway is not involved in the development of gastric-type duodenal adenoma with GDM in non-ampullary lesions. Consequently, total 25 adenomas were divided into 4 gastric-type adenomas (2 foveolar-type adenomas and 2 pyloric gland adenomas) and 21 intestinal-type adenomas.

**Figure 3:**
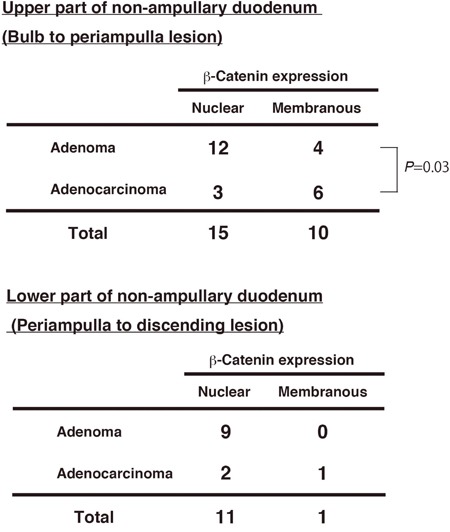
β-Catenin expression and tumor position in non-ampullary adenomas and adenocarcinomas Numbers of nuclear or membranous β-catenin expression in adenomas and adenocarcinomas in “Upper part” and “Lower part” of non-ampullary duodenum except the ampulla of Vater.

Next, we investigated the β-catenin expression pattern in different locations of adenomas and adenocarcinoma in the duodenum. We divided the part of non-ampullary duodenum; the upper (proximal) part from the bulb to the periampulla and the lower (distal) part from the periampulla to the descending duodenum. Nuclear β-catenin was frequent in adenoma and adenocarcinoma of the lower part and adenoma of the upper part (Figure 3). However, in the upper part, adenomas and adenocarcinomas with membranous β-catenin accounted for 4 (25%) of 16 adenomas and 6 (66.7%) of 9 adenocarcinomas, respectively; this difference was statistically significant. Our data indicates that a subset of sporadic adenocarcinomas in the proximal duodenum might be independent of the Wnt/β-catenin pathway, while a subset of adenocarcinomas in the proximal duodenum is associated with the Wnt/β-catenin pathway as well as the distal duodenum.

### ALDH1-positive cells showed significantly increased adenocarcinoma compared with adenoma

**Figure 4:**
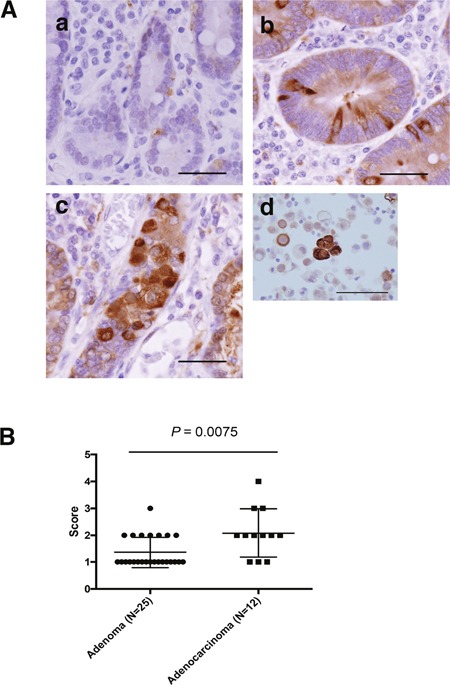
ALDH1 expression as assessed by IHC in non-ampullary duodenal adenomas and adenocarcinomas **(A)** Representative photos of non-ampullary duodenal lesions. **(a)** Normal epithelium. **(b)** Adenoma. **(c)** Adenocarcinoma. Bars: 100 μm **(d)** Lung cancer cells as a positive control of ALDH1 staining (Roudi R, Cancer Invest. 2015). Bar: 50μm. **(B)** Data for ALDH1 expression scores (1 to 4) in adenomas and adenocarcinomas. (*See also Materials and Methods*).

We investigated ALDH1 expression in non-ampullary duodenal adenoma and adenocarcinoma by IHC analysis. ALDH-positive cells were not detected in normal duodenal epithelium; however, ALDH1-positive cells were observed in adenomas and adenocarcinomas (Figure 4A). ALDH1 protein showed predominantly cytoplasmic expression in the tumor cell. Further, we analysed the signal intensity scores of ALDH1 immunostaining in adenoma and adenocarcinoma. The signal intensity was significantly higher in adenocarcinomas than adenomas (Figure 4B), indicating that ALDH1 may associated with the progression from adenoma to adenocarcinoma in non-ampullary lesions.

### ALDH1-positive tumour cells were frequently co-localized with nuclear β-catenin in adenoma

**Figure 5:**
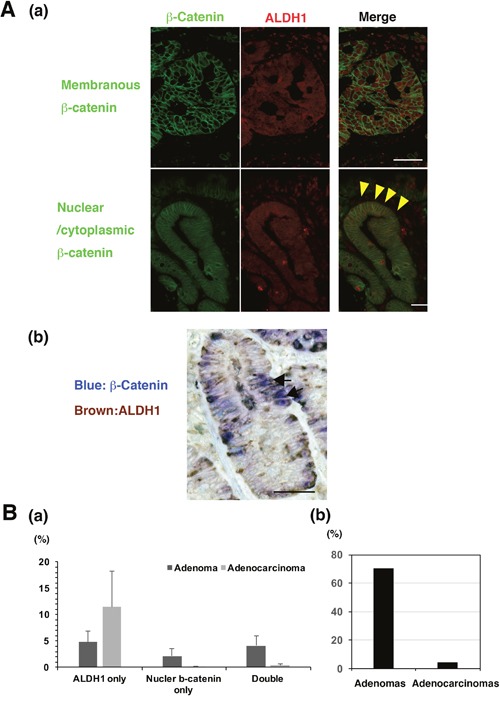
The co-expression of β-catenin and ALDH1 in non-ampullary adenomas and adenocarcinomas **(A) (a)** Double immunofluorescent staining of β-catenin (green) and ALDH1 (red) in representative adenomatous lesions (adenocarcinomas) in the non-ampullary duodenum. *Top panels*: membranous staining of β-catenin. *Bottom panels*: nuclear staining of β-catenin. Arrowheads (yellow) indicate that nuclear β-catenin (green) is co-localized with ALDH1 (red). Bars: 50 μm. **(b)** Representative double-staining IHC of β-catenin (blue) and ALDH1 (brown) in a representative adenomatous lesion (adenoma) in the non-ampullary duodenum. Arrows indicate that nuclear β-catenin (blue) is co-localized with ALDH1 (brown). Bar: 50 μm. **(B) (a)** Showing percentages of ALDH1 only, nuclear β-catenin only, or double positive cells per all the tumor cells of 5 independent fields in non-ampullary duodenal adenoma and adenocarcinoma. Data shows means ± SD. **(b)** the positivity of nuclear β-catenin-positive cells in total ALDH1-posive cells of adenomas and adenocarcinomas

To investigate whether Wnt/β-catenin and ALDH1 activation occur in the same tumor cells of adenoma and adenocarcinoma, we performed both IF and IHC staining for distinct conclusion. In the IF assay, the co-localization of nuclear β-catenin and ALDH1 strong expression was observed in tumor cells of adenoma and adenocarcinoma (Figure 5A). In the IHC assay, we could more clearly detect the co-expressed cells. To evaluate the number of nuclear β-catenin- and/or ALDH1-positive cells, we calculated the number of only ALDH1-positive, only β-catenin-positive, or double positive cells in all the tumor cells of independent 5 fields. The percentage of nuclear β-catenin- and ALDH1-positive cells in tumor cells were predominantly co-expressed in adenoma compared with adenocarcinomas (Figure 5B). In adenomas, only ALDH1-positive cells, only nuclear β-catenin-positive cells, and double-positive cells of nuclear β-catenin and ALDH1 were 4.83%, 2.15%, and 4.14%, respectively, in the region in which nuclear β-catenin was observed most frequently. Inadenocarcinomas, only ALDH1-positive cells, only nuclear β-catenin-positive cells, and double positive cells were 11.5%, 0.06%, and 0.41%, respectively. Further, the positivity of nuclear β-catenin-positive cells in total ALDH1-posive cells between adenomas and adenocarcinomas, resulting that the positivity in adenomas was much more than in adenocarcinomas (Figure 5B). These data suggest that Wnt/β-catenin signalling pathway might be associated with CSCs marked by ALDH1 in adenoma rather than adenocarcinoma.

## DISCUSSION

**Figure 6:**
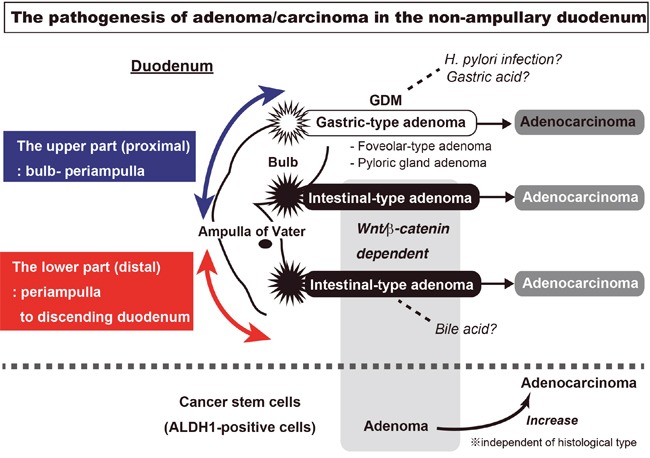
The visualization of sporadic adenoma and adenocarcinoma pathogenesis in the non-ampullary duodenum In non-ampullary lesions of the duodenum, Wnt/β-catenin pathway is associated with intestinal type adenoma in the proximal and distal duodenum, while the pathway was independent of gastric type adenoma in the proximal duodenum. The gastric type adenoma has two subtypes: gastric foveolar-type and pyloric gland adenoma. The gastric type adenoma is associated with GDM (gastric duodenal metaplasia), which may be injured from gastric acid or a *Helicobacter pylori* infection. In the intestinal type adenoma, Wnt/β-catenin activation occurs as well as in the majority of colon carcinogenesis. The ALDH1-positive cells, a putative cancer stem cell marker, is more expressed in adenocarcinoma than adenoma. However, the co-localization with nuclear β-catenin in adenoma is more frequent than adenocarcinoma.

In this study, we clarified the expression of β-catenin and ALDH1 in sporadic adenomas and adenocarcinomas in non-ampullary duodenal lesions by IHC. We have suggested the pathogenesis in sporadic non-ampullary duodenal adenoma and adenocarcinoma(Figure 6). We demonstrated that the Wnt/β-catenin pathway was associated with intestinal type adenoma in the proximal and distal duodenum, while the pathway was independent of gastric type adenoma in the proximal duodenum in non-ampullary lesions.

We did not include any cases of ampullary duodenal adenomas or adenocarcinomas because ampullary neoplasms may have combined features of neoplasms of the small intestine including duodenum, distal bile duct, and/or pancreas. Adenocarcinomas are particularly rare in non-ampullary lesions compared with adenomas.

Nuclear β-catenin accumulation, a key effector of the Wnt signalling pathway, was frequent in adenoma rather than in adenocarcinoma. There were more ALDH1-positive cells with CSC potential in adenocarcinomas compared to adenomas. Further, nuclear β-catenin in these cells was more frequently co-expressed in adenomas rather than in adenocarcinomas. Our data suggest that Wnt/β-catenin activation might be associated with ALDH1-expressing CSCs in a subset of non-ampullary duodenal adenomas, i.e. intestinal type adenomas. There are similar findings in breast cancer, where ALDH1-positive cells show higher levels of Wnt/β-catenin signalling compared with ALDH1-negative cells [24]. By contrast, in adenocarcinomas of the proximal duodenum, Wnt/β-catenin activation was less frequent and ALDH1-positive cells were more frequent than adenoma. This result may be caused by the development of diverse tumors in the proximal duodenum compared with the distal duodenum. Adenocarcinomas are difficult to categorize due to their heterogeneity, but adenomas are easily categorized into intestinal type, gastric foveolar type, and pyloric gland adenomas.

Our study showed nuclear β-catenin expression in 84% of adenomas and 33.3% of adenocarcinomas in non-ampullary duodenal lesions. A previous study observed aberrant β-catenin expression and nuclear/cytoplasmic translocation in 38.1% of duodenal adenocarcinomas, including both non-ampulla and ampulla of Vater [37]. In contrast to our results in non-ampullary lesions, 8.2% of adenomas and 40.5% of adenocarcinomas showed nuclear β-catenin expression in ampulla (of Vater) lesions [38]. Alterations in β-catenin may occur late in neoplasm carcinogenesis in the ampulla of Vater. However, our data suggests that in non-ampullary lesions, alterations in β-catenin might be an early event.

In contrast to the typical adenoma-carcinoma sequence in the majority of colorectal carcinogenesis, the development of adenocarcinoma in non-ampullary duodenal lesions seems to have two types; 1) *de novo* type, i.e. gastric type, that occurs independent of the Wnt/β-catenin pathway, 2) adenoma-carcinoma sequence type, i.e. intestinal type, that develops dependent of the Wnt/β-catenin pathway like colon carcinogenesis. A possible difference between the non-ampullary duodenum and colorectum can be observed in the developing gut. Developmentally, the duodenum appears to share characteristics with the stomach, particularly the antrum, rather than the colorectum [39]. In our study, all the gastric-type adenomas with GDM developed in the proximal duodenum, and were associated with membranous β-catenin. This suggests that the non-ampullary proximal duodenum is likely to have gastric characteristics injured by the environment, such as gastric acid and *H. pylori*.

Regarding colorectal cancers, the CSC population detected using ALDH1 enzymatic activity is typically very small, including 0.01–1% of the population of cancer cells [40]. In contrast, immunohistochemical studies examining various types of cancers have reported remarkably high fractions of positive cells [41, 42]. Only one immunohistochemical study has examined small intestinal adenocarcinoma, and strong ALDH1 expression was observed in 33.3% of all cases [43]. In our study of non-ampullary lesions, ALDH1-positive cells occurred more frequently in adenocarcinomas than in adenomas, and high ALDH1 expression (Score 3 and 4) was observed in 33% (3/9) of adenocarcinoma cases. This is similar to a colon adenocarcinoma study where ALDH1 positivity in IHC experiments demonstrated an increasing number of colonic stem cells over colon cancer progression [23]. Thus, the ALDH1-based CSC marker can be used to track CSCs in the sporadic non-ampullary duodenal adenoma/adenocarcinoma during the tumorigenesis as well as colon tumorigenesis.

In conclusion, nuclear β-catenin accumulation is more correlated with adenomas rather than adenocarcinomas in the non-ampullary proximal duodenum. The co-localization of nuclear β-catenin and ALDH1 is common in adenoma, and ALDH1 is highly expressed in adenocarcinoma rather than in adenoma. The pathogenesis may differ in sporadic adenoma and adenocarcinoma of the non-ampullary duodenum, especially the proximal and distal duodenum.

## MATERIALS and METhODS

### Patient and tissue samples

Tumor specimens were obtained from patients through biopsy, EMR, or ESD at Gifu University Hospital between 2008 and 2015, and Gifu Municipal Hospital between 2012 and 2015. Informed consent was obtained from all patients.

### Diagnosis of adenoma and adenocarcinoma

Using hematoxylin and eosin-stained sections, adenoma was diagnosed when the tumor was circumscribed and found to be composed of tubular structures showing intraepithelial neoplasia. Adenocarcinoma was diagnosed when the tumor invaded into the lamina propria or through the muscularis mucosae. On biopsy, samples contained areas suggestive of true invasion. Adenocarcinoma without invasion was diagnosed when increased architectural distortion with glandular crowing and prominent cellular atypia and pleomorphic, hyperchromatic, pseudostratified nuclei were observed. The diagnoses were confirmed by at least two pathologists.

### Immunohistochemical staining

All paraffin-embedded tissues were cut into 4 μm-thick serial sections and deparaffinized. These sections were stained with hematoxylin and eosin or used for IHC. For IHC, the sections were placed in citrate buffer (pH 6.0), and then autoclaved at a chamber temperature of 121°C for 1 min to retrieve the antigen. The sections were then rinsed and blocked in 3% hydrogen peroxide in methanol for 10 min to remove endogenous peroxidase. Non-specific binding sites were blocked in 0.01 M phosphate-buffered saline (pH 7.4) containing 2% bovine serum albumin (Wako Pure Chemical, Osaka, Japan) for 40 min. They were incubated with β-catenin antibody (Mouse IgG, 1:500; BD Transduction Laboratories, San Jose, CA, USA) and ALDH1A1 antibody (Rabbit IgG, 1:500; Abcam, Cambridge, UK) overnight at 4°C.

Primary antibodies were detected using a biotinylated anti- mouse and rabbit IgG (1:300; Dako, Glostrup, Denmark) for 30 min at room temperature, followed by incubation with avidin-coupled peroxidase (Vectastain ABC kit; Vector Laboratories, Burlingame, CA, USA) for 30 min. The bound complex was visualized using diaminobenzidine liquid chromogen (Sigma, St. Louis, MO, USA), and counterstained with hematoxylin. As a negative control, the primary antibody was replaced with normal serum IgG at a similar dilution.

For double-staining IHC, β-catenin and ALDH1 were used as primary antibodies. Signal amplification was performed using alkaline phosphatase-biotin complex (Vectastain), followed by a chromogenic reaction with an alkaline phosphatase substrate kit (Vecta Blue; Vector Laboratories) and diaminobenzidine.

For immunofluorescent (IF) staining, after treatment with primary antibodies, samples were incubated for 30 min at room temperature with FITC- or rhodamine-conjugated secondary antibodies (1:500 dilution; Dako). DNA was labeled with 4′, 6-diamidino-2-phenylindole. Histological evaluation was performed by two experienced pathologists (H.T. and A. N.), who were blinded to the clinical data.

### Assessment of β-catenin and ALDH1A1 immunostaining

Staining of β-catenin was graded as either nuclear (moderate or strong staining of the nucleus) or membrane (very weak or negative in the nucleus). ALDH1 staining intensity scores were rated on a scale of 0–4 according to the percentage of positive tumour cells (0, < 5% positive cells; 1, 5–10%; 2, 11–30%; 3, 31–50%; 4, >50%).

In double staining, percentages of nuclear β-catenin- and/or ALDH1-positive cells in adenoma and adenocarcinoma were obtained by manually counting positively stained cells in 10 representative fields of adenomatous or cancerous regions, in which nuclear β-catenin translocation was most frequently observed under 400× high-power magnification.

### Statistical analysis

Statistical analysis and graphical presentation were performed using the GraphPad Prism 6.0 package (Graph-Pad, San Diego, CA, USA). The Mann-Whitney U, Fisher's exact test, or chi-square test was used to test the relationship between categorical variables as appropriate. *P* < 0.05 was considered to indicate statistical significance.

## SUPPLEMENTARY MATERIALS FIGURES AND TABLES


