# Correction: The different pathogenesis of sporadic adenoma and adenocarcinoma in non-ampullary lesions of the proximal and distal duodenum

**DOI:** 10.18632/oncotarget.24518

**Published:** 2018-02-16

**Authors:** Ayumi Niwa, Seiya Kuwano, Hiroyuki Tomita, Keita Kimura, Yukiya Orihara, Tomohiro Kanayama, Kei Noguchi, Kenji Hisamatsu, Takayuki Nakashima, Yuichiro Hatano, Akihiro Hirata, Tatsuhiko Miyazaki, Kazuhiro Kaneko, Takuji Tanaka, Akira Hara

**Affiliations:** ^1^ Department of Tumor Pathology, Gifu University Graduate School of Medicine, Gifu, Japan; ^2^ Division of Animal Experiment, Life Science Research Center, Gifu University, Gifu, Japan; ^3^ Division of Pathology, Gifu University Hospital, Gifu, Japan; ^4^ Department of Gastroenterology, Endoscopy Division, National Cancer Center Hospital East, Kashiwa, Japan; ^5^ Department of Diagnostic Pathology (DDP) and Research Center of Diagnostic Pathology (RC-DiP), Gifu Municipal Hospital, Gifu, Japan

**This article has been corrected:** the title has been corrected.

Original article: Oncotarget. 2017; 8:41078-41090. https://doi.org/10.18632/oncotarget.17051

