# Supplementary material for: The different pathogeneses of sporadic adenoma and adenocarcinoma in non-ampullary lesions of the proximal and distal duodenum
Source: Oncotarget. 2017 Apr 12;8(25):41078–90. doi: 10.18632/oncotarget.17051 (PMC5522249; doi:10.18632/oncotarget.17051)
Supplement: Supplementary file 1 [file oncotarget-08-41078-s001.pdf]

# The different pathogeneses of sporadic adenoma and adenocarcinoma in non-ampullary lesions of the proximal and distal duodenum

## SUPPLEMENTARY MATERIALS

## SUPPLEMENTARY FIGURE

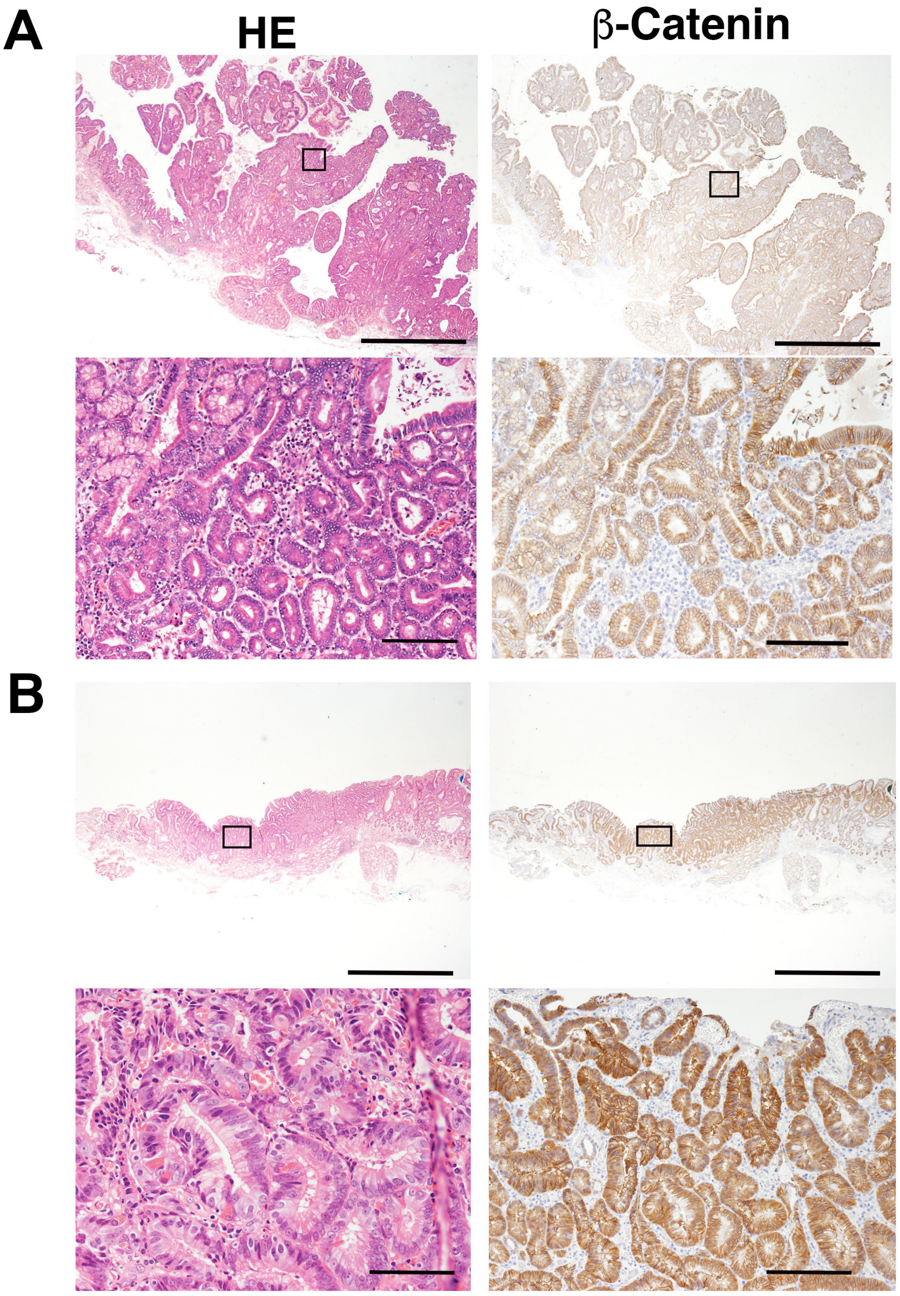


**Supplementary Figure 1: β-Catenin expression in representative duodenal adenocarcinomas.** **(A)** membranous β-catenin **(B)** Nuclear and cytoplasmic β-catenin each inbox indicate lower photo. Bars, upper panels; 500µm, lower panels, 20µm.
